# Knowledge on human papilloma virus and experience of getting positive results: a qualitative study among women in Kilimanjaro, Tanzania

**DOI:** 10.1186/s12905-023-02192-8

**Published:** 2023-02-11

**Authors:** Patricia Swai, Melina Mgongo, Beatrice J. Leyaro, Julius Mwaiselage, Bariki L. Mchome, Susanne K. Kjaer, Vibeke Rasch, Rachel Manongi, Sia E. Msuya

**Affiliations:** 1 Department of Obstetrics and Gynaecology, Kilimanjaro Christian Medical University College (KCMUCo), Moshi, Tanzania; 2 Department of Obstetrics and Gynaecology, KCMC Hospital, Moshi, Tanzania; 3 Department of Community Health, Institute of Public Health, Kilimanjaro Christian Medical University College (KCMUCo), Moshi, Tanzania; 4 Department of Epidemiology and Biostatistics, Institute of Public Health, Kilimanjaro Christian Medical University College (KCMUCo), Moshi, Tanzania; 5 Ocean Road Cancer Institute, Dar es Salaam, Tanzania; 6 Department of Gynaecology, Rigshospitalet, Copenhagen University Hospital, Copenhagen, Denmark; 7 Department of Obstetrics and Gynaecology, Odense University Hospital, Odense, Denmark; 8 Department of Clinical Research, University of Southern Denmark, Odense, Denmark; 9 Department of Community Medicine, Kilimanjaro Christian Medical Centre (KCMC), Moshi, Tanzania

**Keywords:** HR-HPV, HPV results, HPV screening, Screening, Reaction, Tanzania

## Abstract

**Background:**

Human papilloma virus (HPV) is a sexually transmitted infection causing more than 80% of cervical cancers. WHO recommends using of sensitive screening methods like HPV-testing to timely prevent future morbidity and mortality from cervical cancer. Pilot studies have shown that HPV-testing is feasible and can be scaled in developing country like Tanzania. However, there is limited information on women understanding, reactions and psychological challenges following diagnosis of high risk HPV (HR-HPV). This study explored the knowledge of women on HPV and their experience after HPV positive results in Kilimanjaro, Tanzania.

**Methods:**

The study was part of a larger study that assessed incidence and persistence of HR-HPV among women aged 18 years and above in Kilimanjaro. This was a cross sectional study conducted in Moshi municipal council among women who had HR-HPV positive results at enrollment. In-depth interviews were conducted with 13 randomly selected women who were attending for follow-up after enrollment. Interviews were conducted at the health facility and Atlas.ti.8 was used to analyze the data using thematic framework analysis.

**Results:**

Women had knowledge on HPV infection but they had different reactions following receiving positive HPV results. Reaction toward the positive HPV results had two extremes; some women had psychological effect (hopeless, death sentence, having cancer, being shocked, failure to disclose and psychosexual effects) while others women explained positive results is good as they are identified earlier, will be followed up and it has made them plan to continue with cervical cancer screening in future.

**Conclusion:**

Women had knowledge on HPV, but positive results lead to negative and positive experiences by women. Clinicians and programs need to develop interventions and good strategies to minimize the psychological and social burden of testing positive for HPV.

## Background

Human papilloma virus (HPV) is a group of viruses that are very common worldwide. HPV are transmitted through sexual contact with a person affected with the virus [1]. High-risk human papilloma virus (HR-HPV) causes cervical cancer, which is the fourth most common cancer among women and second common cancer among women in low- and middle-income countries (LMIC). About 90% of the cervical cancer deaths occur in LMIC [2]. Limited access to cervical cancer screening and treatment services in the LMICs is a key reason for higher number of cases and deaths [3, 4]. The incidence of cervical cancer in high-income countries (HICs) has been reduced by 70–90% because of the implementation of effective screening programs [3].

As reported by WHO, cervical cancer can be prevented by using HPV-vaccination among young girls. Screening and treatment of pre-cancerous lesion using visual inspection with acetic acid (VIA) in LMIC countries or pap smear in HICs have been the main secondary prevention methods [4, 5]. Screening using HPV methods are increasingly recommended by the WHO and other researchers, in both resource-poor and rich countries. Randomized trials have shown that screening with HPV tests have higher sensitivity and protects better against future cervical precancerous lesions and invasive cancers than previous tests [6–8]. To achieve the goal of eliminating cervical cancer, one of the targets set by 2030 is to screen 70% of women in a particular setting using a high-performance test [1, 2].

Studies from high-income setting where HPV test have been done have reported there are negative effects associated with getting HPV positive results. HPV is transmitted sexually, thus HPV positive results have been shown to affect sexual relationships, sexual satisfaction, and for some it lead into anxiety, disappointment and depression [8]. Some studies on HPV testing in cervical screening suggested that some women with HPV have concerns about disclosing HPV positive test result to their partners due to the feelings of stigma and shame,, worries to receive a negative reaction from their partner, being rejected by their partner, or partner end their relationship [8, 9].

Adequate knowledge of HPV results and screening procedure to women is reported by other scholars that it helps in planning new guidelines and adherence to cervical cancer screening [8, 10]. Tanzania has adopted both primary and secondary prevention methods to decrease cervical cancer incidence. In its cervical cancer strategic plan of 2020–2024, the country planned to introduce HPV testing as an additional method of screening for cervical cancer at the level of zonal and regional hospitals [11]. Pilot testing that was done in Dar es salaam and Kilimanjaro regions using rapid-HPV tests have shown that the test can be performed at all levels of health facilities from primary care clinics to regional hospitals [12].

However, in Tanzania there are limited studies that has explored women knowledge and experiences in getting HPV positive results, thus make the need for this study. This information will help to inform the screening program on what to target during counseling when HPV screening methods for cervical cancer will be used and scaled-up in future.

## Methods

### Aim and study settings

This explorative qualitative study was part of the longitudinal study (CONCEPT project- comprehensive prevention of cervical cancer in Tanzania) that was conducted from August 2015 to October 2018 in Dar es Salaam and Kilimanjaro regions. The CONCEPT project involved cervical cancer screening for all women who participated in routine cancer screening services provided at Kilimanjaro (Mawenzi Regional Referral Hospital and Kilimanjaro Christian Medical Centre -KCMC) and at Ocean Road Cancer Institute (ORCI) in Dar es Salaam. At enrollment women were tested for HIV, HR-HPV, and Pap smear for cytology and were given HIV results on the same day and pap smear and HPV results within a month of testing. The details of the CONCEPT project are described elsewhere [13]. This study uses women who were enrolled in the CONCEPT study in Kilimanjaro and explored how women understood, internalized, and reacted to positive HPV results.

### Study population

The study population for qualitative study were women with high-risk human papilloma virus (HR-HPV) positive results and were enrolled from Kilimanjaro region (n = 336). All women with positive HR-HPV results were phoned to come for their results including HPV results. At the respective clinics (Mawenzi or KCMC), women were given their HPV results, general education on HPV and counseling on HR-HPV and future screening procedure and follow up. Twenty women were randomly selected out of 336 women, and through telephone calls they were invited to return to the clinics two weeks after receiving their results. They were informed about plans for interviews to understand how they are taking the HPV-positive results and their experiences. 13 of the 20 women came for the interviews.

### Data collection

The in-depth interview guide was used for data collection and it had written questions and probes that were adopted from previous literatures [14]. The guide comprised of sociodemographic questions, knowledge on cervical cancer, HPV and screening, the process involved in HPV screening, women experience in getting cervical cancer results especially HPV results, their reaction, perceptions towards sharing results and future counseling plan.

The interviews were conducted by a nurse who is experienced in qualitative research. All the interviews were conducted in Swahili, which is the national language. Confidentiality was discussed before each IDI and numbers were used to identify all participants. All interviews were tape-recorded and qualitative researcher took notes during the interviews. Saturation was reached after conducting 13 out of 20 planned in-depth interviews. A private room for IDI was identified from the cervical cancer clinic separate from the screening room. The in-depth interviews lasted for about 45–50 min.

### Data analysis

Thematic data analysis was performed as per Braun and Clarke [15]. The steps involved familiarization of data whereby the in-depth scripts were transcribed verbatim read and translated to English by the competent Swahili and English speaker. Then followed by development of initial codes by two independent coders (MM, BJL) using ATLAS.ti 8 program. The initial codes were developed independently after reviewing the transcripts and later compared by the authors. In case there was disagreement another coder (PS) joined the team to solve disagreement. The authors agreed on the developed codes, and for any new code that emergent the authors discussed the code and after agreement the codes were added in the code list. The next involved searching for themes and all codes that were related were sorted and listed into one comprehensive theme. After theme development, the last step was reviewing and refining themes and report writing.

## Results

A total 13 women participated in the study, with age range between 28 and 60 years. Majority of women had primary education (9/13), were married (9/13) with two children (Table 1).

**Table 1:** Characteristics of study participants with HPV positive result women (N = 13)

	No	%
*Age (years)*		
25–35	3	23.1
36–46	8	61.5
47–60	2	15.4
*Education level*		
Primary	9	69.2
Secondary	2	15.4
University	2	15.4
*Marital status*		
Married	9	69.2
Single/divorced	4	30.8
*Parity*		
0–2	9	69.2
3–4	3	23.1
5+	1	7.7

A total of four themes were identified; knowledge on HPV and screening process, reactions towards receiving positive results, sharing of HPV results and recommendations to improve uptake of cervical cancer screening to women.

### Knowledge on HPV and screening process

In this study women had knowledge about HPV. They mentioned that HPV is a virus that causes cervical cancer, and all women are eligible to get tested for HPV. “Virus that can cause cervical cancer and can also cause other diseases” *ID 1*. They further mentioned HPV is transmitted through sexual intercourse and it is risk if a woman has sexual engagement with different men. “This virus enters the body through sex with more than two, three men” *ID 13*.

The women mentioned that screening helps to identify if the woman has early signs of cervical cancer, which in turn helps to get early treatment.

“They test the uterus, the cervix, to see if there is a problem and if there is any early problem, they tell you and they see in what way they can help on the early stage” *ID 12.* Though majority of women were aware of HPV, only three women were aware of the benefits of cervical cancer screening. Majority of women mentioned the HPV screening process was friendly and women were able to demonstrate the process. “They tested the cervix, they took the mucus and said it’s going to be tested and you will be given the results later on” *ID 6*.

Though women mentioned to have received better services in the screening process, two women had a concern that there should be increased privacy. Some women were scared and felt ashamed when asked that there will be investigation in their private parts and did not feel comfortable having a male doctor.

Despite having knowledge on HPV, the concept of persistence of the HPV virus was confusing. The women acknowledged that the health care providers informed them that the virus might persist or disappear after sometimes, but this created tension as explained in the narrative below.

“It is scaring not knowing if am among the group where the HPV can persist, and I will end with cancer or not. It is very stressful I can tell you, however much they counsel and comfort you” ID 2.

### Women reactions towards receiving HPV positive results

#### Duration and result giving process

Majority of women voiced that the process of getting result was not good. They mentioned that the health care informed them that for those who would have positive results, they will be called to come to the facility after two weeks. Majority of women become anxious and kept on thinking what will be their results and for those who forgot the process it was a shock to receive a call from the facility.

“They phoned, when I went is when they explained to me that the test that was taken was found positive, I told them positive does it mean I have cancer? They said no it’s not cancer but you have the pathogens that can cause cancer. I asked them what I should do, are you giving me medicine or removing the uterus or what are you doing to me? They told me no, your immunity will clear that thing but you have to come and check again after a year. So I returned having stress” IDI 3.

“Let’s say like the way I received them honestly if had pressure I would have fallen down; I thought my goodness this is death” ID 4

After reaching the health facility, women mentioned that the health care providers did not provide much information about the results thus left with many unanswered questions as explained in the narratives below.

“My Doctor should have explained it more to me to understand because I was left with many questions of which I did not have answers” IDI 1. Participant continues “what will I do with this virus, is there no medicine, and when you are that way how long will you live then die, are there other effects, things like that. I know the results are there and he would have not be able to change them” IDI 1.

#### Receiving positive results

Women had different reactions after knowing that they had a positive HPV result. Some thought the results as a death sentence or end of life. “Personally, first it frightened me because I saw that this might be the end of my life, to be honest I was afraid, I was very afraid”* IDI 12*. Other thoughts the results meant they have cancer as narrated by few participants “In the beginning when I was told of course I lost hope. Because I thought I already have cancer”* IDI 8. *“They phoned, when I went is when they explained to me that the test that was taken was found positive, I told them positive does it mean I have cancer?* IDI 3. *“Honestly it was not a good day completely because first I never knew there was such a disease, I just know cancer, when they gave me those results honestly I was shocked, I knew death is a must”* IDI 16.* Some women counseled their plans to have children thinking it might cause disease progression as narrated here “I wanted later on to conceive but with positive results I do not want children anymore, I have cancelled those plans”* IDI 2.*

“You know I have completed a year and if it’s a year I would have already had a baby but those are the issues that have made me to still wait and you know….” IDI 3

But after receiving counseling it helped them to calm down and focus on the advice that was provided at the health facility.

“I received them with little uneasy but after the educator explaining it to me, I realized it’s a normal thing and has no problem in my body” IDI I0.

In some situation women felt guilty to have had sex, *“*when I remember I feel so bad, as you know from people stories they say this thing comes from men, so I thought if I did not have sex it means I would have not had this thing and after the results I did not have much interest for sex” IDI 2.

The fear due to receiving positive HPV results made another woman to go for second test, and she was still anxious about the results. “Yes honestly it gave me thoughts to the point that I had another test done there and I am afraid to ask for results because if I will be told again its positive it means the immunity has not work and how will I know if I go after a year it will have worked? So honestly it’s stressful” *IDI I3*.

However, few women had positive opinion to the positive results. The women mentioned that having positive HPV results gave them hope and future plan for cervical cancer screening. “Honestly I was very happy to be checked my health, so happy that I have known I have a problem that when one day I fall sick will fail to know when the problem had start” *IDI 6.*

### Sharing of HPV results

#### Person to share results

Majority of women were able to share their HPV results to their closest family members mostly their partners, mothers, and sisters. Women trusted their mothers as the first person to share their results then the husbands or partners.

“Yes, the first person to disclose my results of HPV-test was my mother. She had already received this education… she was aware, she just said it has no problem and advised me to tell my partner because he is the closest person to me. So, from there is when I decided to disclose it to my husband” IDI 8.

Another woman was also positive to share results as narrated herein “yes I saw there was a need to share with someone. First, I shared with my husband and explained to him well and he understood that I don’t have cancer but have just the virus, so he told me don’t worry you don’t have anything. Later on, I shared with my other relative who is my sister and she told me you don’t have to worry because that thing is not there and it just things that are in the body and will end on its own time*”* *IDI 12.*

#### Fear of disclosing results

In this study three mothers did not want to share their HPV results to anyone. Women had a fear that sharing results to others might cause them to be labeled as cancer patients. They also feared HPV being an STI; they might be seen prostitutes by their partners, so they thought there is no need to cause marital problems. They further mentioned that the results are personal and should not be shared with another person. If there is a problem, they will find a way to deal with it.

“I thought if I tell him he is going to get stress, he will get a lot of stress….I decided to keep it to myself and deal with it my way/ die with it” IDI 3

Another participant mentioned to have not shared the results but if she has to share she will share to her relative “maybe if it happens one day I decide to explain it to my relative or to,,,, mostly my relative” she continues explaining that the results caused anxiety and she puts everything in Gods hand “I was worried but I left it to God and said if it’s to die I will die normally like any other person and human being must die, but if this problem is discovered early and is treatable I will be helped” *ID 4.*

#### Concerns from closest family members

Sharing positive HVP results created tension to their family members. The relatives thought that women had cancer and their husband did not understand the meaning of results. However, women were insisted with their relatives to make regular check up to the hospital and in some situation their partners planned to visit the health facility to get more clarifications.

“I shared with my husband, I explained to him and he was shocked but I calmed him by saying it’s not cancer and it is something that can stay in the body for several years and go away on its own or cause cancer, so what he advised me is to continue with follow up and not neglect and cause problem” IDI 13.

One woman is this study mentioned to have received encouragement from her husband and her mother after having a positive HPV result.

“Yes, I saw there was a need to share with someone, first I shared with my husband and explained to him well and he understood that I don’t have cancer but have just the virus, so he told me don’t worry. Later on, I shared with my sister” ID 12

### Recommendations to improve uptake of cervical cancer screening to women

Women proposed different ways that can be used to improve the whole process from screening to giving results and increase the uptake of HPV screening to the community at large. Women suggested that when giving results at least they should be phoned to attend clinic and given results face to face. They also suggest that face to face will help them have a discussion with doctor and being able to ask questions that they have in mind. It was voiced that it is important for the same doctor who did examination to provided results to avoid inconveniences that cause women to be shocked.

“To another person I would like after doing the test, they call you and give you the results when you are face to face with your doctor. They should try to use words that will make a person come without fear and the doctor who did the test should explain to you face to face, your results” IDI 1.

Women also mentioned that it is important to bring services to the community like doing home visit to sensitize women and make services close to the community as each ward has a health center.

“Would advise that the health providers to pay visits in the communities in streets, because there are people who are lazy going to the hospital claiming that it takes a long time to wait for the service. So I advise them to arrange even for home visits, it will be nice as everyone will be able to reach their service. Another option is to bring this service close to the community like having these services to the facilities in every ward as every ward has a health facility, which would also been easy. ID 7.

## Discussion

In this study, majority of women had knowledge about HPV infection, despite having good knowledge women had different reactions towards the screening process, getting HPV positive results and also suggested ways to improve the testing process and recommendations to improve uptake of cervical cancer screening to women.

Women stated that the HPV is sexually transmitted and screening helps to identify if they have problem and seek medical treatment early. After receiving HPV positive results, women had to consult theirs doctors for further clarification and knowledge on HPV. This is in line with the study from Australia whereby women who tested positive for HPV had more knowledge as they further had search for knowledge on HPV [16]. Having knowledge on HPV will improve cervical cancer screening and follow up to reduce morbidity and mortality resulting from cervical cancer, hence eliminate cervical cancer by 2030 [17]. Thus implementing HPV screening methods need to go hand in hand with good explanation on HPV occurrence, consequence and importance of follow up [18].

Some women expressed fear, shame, stress and anxiety after receiving HPV positive results. In this study women reported that it is difficult to share HPV positive results with their spouses as HPV is sexually transmitted disease and this could bring conflicts to their marriages i.e., being labeled as prostitutes, and may end up being divorced. On the other hand it has previously reported that women has financial dependence from their partners [19] so being separated/divorced might cause financial challenges to them.

These findings are similar from report done in Ireland and United Kingdom [18, 20].

The results of our study implies that the disclosure is challenging and this can lead to spread of the virus if the partners engage sex with others as it has been reported from other studies [8, 16, 21].

Many women who had positive HPV results thought that they already have cervical cancer, or will develop cervical cancer in near future which gave feelings of hopelessness, fear and anxiety. Understanding the meaning of HPV infection, test and result interpretation will make women and community understand being positive for HPV is not synonymous with having cervical cancer [20]. This observation is very important for cervical cancer prevention program in the country as it intends to add HPV screening in routine screening. They need to develop simple yet comprehensive education materials for women and the community on HPV, its natural progression, probability of body clearing the infection, probability of persistence and why there is a need for future follow-up and screening [11, 13].

In this study for some women positive HPV results influenced reproductive choices. Some women who tested positive for HPV changed or cancelled their plans to have children in their future. Women had fear that if they will be pregnant their pregnancy will increase risk for cervical cancer and their babies will be at risk if their future lives. The results are similar to study report from Australia whereby women cancelled the need to have children after receiving positive results [16]. The result of our study means that there is a need to educate and counsel women when receiving HPV results, thus will reduce myths about testing HPV positive in relation to bearing children.

In this study participants reported to have different concerns from healthcare providers, the concerns included lack of privacy and not getting information from the questions that they had. These findings imply that the participant might talk negatively about the HPV screening process and affect the uptake of the intervention in early identification of the cases. Other studies have reported that inadequate knowledge to the health care providers affects uptake of the intervention [22]. The results of this study call for a future study to assess heath care workers knowledge and support they provide to women diagnosed with HPV.

### Strength and limitations

This was the first study based from low income countries were HPV is still not well explored since the initiation of the vaccine, and to our knowledge no studies have been done trying to explore the inner filling of testing and receiving HPV results. As WHO and some developed countries have started to implement the new test for cervical cancer screening, our countries are still practicing VIA. Tanzania is planning to implement HPV testing in near future hence knowing how they perceived the results would be good for use in counseling guideline plans. The study on reactions towards HPV testing was only done among women with HPV- positive results, leaving women with HPV-negative results, understanding perceptions in this groups maybe different and important in formatting counseling and education information. Large study involving partners to give their inner insight would help in brooder picture how to develop guidelines with all answered questions from two sides as the disease is sexually transmitted.

## Conclusion

While women understood the HPV and HPV positive results and many understood that they will need to come for check-up after given time. The results however were accompanied with fear of having cervical cancer, despair and anxiety. For others results affected their sexual life and future plan for children. Fear also stemmed from not knowing whom the HPV-virus will persist and lead to cancer. Clinicians and programs need to develop interventions and good strategies to minimize the psychological burden of testing positive for HPV aiming at avoiding unnecessary harm to women taking part in cervical screening. Use of clear, evidence-based communication guide during explanation for test, procedure and result and all information materials like leaflets/ brochures will help ensure that women understand their results and the implications for cancer risk.

## Data Availability

The data used during the current study is available from the corresponding author on reasonable request.

## Abbreviations

CONCEPT: Comprehensive prevention of cervical cancer in Tanzania
HIC: High-income countries
HIV: Human immuno deficiency virus
HPV: Human papilloma virus
HR-HPV: High risk human papilloma virus
IDI: In-depth interview
KCMC: Kilimanjaro Christian Medical Centre
LMIC: Low and middle income countries
ORCI: Ocean Road Cancer Institute
SSA: Sub Saharan Africa
STI: Sexual transmitted infections
VIA: Visual inspection with acetic acid
WHO: World Health Organization
